# The Jamaica Physical Journal

**Published:** 1834-10-01

**Authors:** 


*The Jamaica Physical Journal.* Edited by JAMES PAUL, Esq. M.R.C.S. of London, &c. Nos. I.—VI., from January to June, 1834.—Kingston. 8vo. pp. 392.

THIS is a well-conducted periodical, and promises to be of service to our brethren in the West Indies. It consists partly of articles judiciously selected from European and American Journals, and partly of original papers. Among the former we find several reviews, taken from our first Number, and among the latter some interesting cases, with one of which we will gratify our readers. It is narrated by the editor himself, and is entitled "Case of Recovery after severe Wounds."

"Sam, a negro, belonging to New-Pera estate, St. Thomas in the east, was addicted to running away, and consequently to plundering the provision-grounds in the neighbourhood, which he had successfully accomplished more than once on the neighbouring plantation Fairfield. The watchman of the latter place, finding his efforts to prevent the frequent depredations of no effect, was determined to watch his next appearance, and no doubt intended, if possible, effectually to put an end to the system; for which purpose, with cutlass ready in hand, he crouched by the side of the fence, not far distant to the aperture he knew the thief would pass. He had not long taken his station when the marauder appeared, who, unconscious of his intended reception, glided carefully along to the plantain-trees whose fruit was ready to be cut down. When he thought he had enough, he 'bagged his game,' and, throwing his load over his shoulder, was making for the place at which he entered: it was now that the watchman considered he had the opportunity of claiming his prisoner, and at once pounced upon him. Sam was not however so easily to be caught; and, watching the approach of his opponent, when he thought he was within reach, mustered all his force, threw his burden at him, and then took to his heels. As soon as the watchman recovered himself, he started in pursuit, and, before he gained the outlet, he was within stroke of his cutlass, which he immediately began to use. Before he was brought to the ground, he had received a sweeping cut, from the neck down the back, to the left of the spine, in length five inches, but only through the skin, a wound on the occiput, two inches in length, penetrating the outer table of the skull; and another over the posterior portion of the left parietal, which, glancing off, denuded about two inches square, the flap hanging by its lower edge; it was in all probability the former which had the effect. When he fell, he had quickly turned upon his back, probably for the purpose of grappling with his pursuer, who, taking advantage of his prostrate state, stood over him, and used his weapon with right good earnest, first right and then left, until he found there was no longer a chance of escape, or, more probably, that he had finished his man. He however left the poor wretch on the ground, went to the overseer of the plantation, and related what he had done. Additional people were then sent to the place, who conveyed him to the hospital; and I was sent for in the morning. I found him very weak, unable to speak aloud, and no pulse at either wrist. He had lost a large quantity of blood, but it had stopped, and there was not now the least oozing. His wounds lay gaping, and a cadaverous smell issued from them. On the arrival of Dr. Wm. Pine, who I

requested to assist me, we proceeded to examine the wounds, and determine what was to be done. Besides those already enumerated, both arms had the same quantity, and corresponding in situation, from the shoulder to the little finger. The deltoid muscles on both arms were cut through, the capsular ligament of the shoulder-joint laid open, and the head of the bone fractured. Spiculæ of bone were extracted from both joints; the next wounds were midway between the shoulder and elbow; they were mere fleshy wounds. Betwixt the elbow and wrist were three on each arm, also fleshy, except the carpal one on the right arm, which severed both bones, leaving the hand dangling by the flexor tendons on the fore part of the arm; the little finger of the left hand was split its whole length; there was a stab in the right side, and the little toe of the left foot was cut off.

"The debilitated state of the man precluded the possibility of operating, and we were at a loss to make up our minds to leave the right arm, where there appeared so little probability of a successful termination. We however judged that the most prudent course would be delay. All the wounds were therefore dressed, bringing the edges of each together by adhesive straps, and, where required, by sutures: the right fore-arm was treated as a compound fracture, the ends of the bones being placed in juxta-position, retained there by splints, and the whole bandaged. Gave him an opiate, and ordered gruel to be given occasionally. The following day he said he felt revived: the pulse was distinguishable at the left wrist; his skin had regained a natural warmth, and altogether he seemed to be doing well. Two days after, the wounds began to discharge, and, upon taking off the dressings, was agreeably surprised to see their very healthy appearance. Nothing remarkable now occurred till about a month after, when he was attacked with measles, with which he suffered severely, having no use of his arms, and consequently being unable to raise himself from a horizontal position, so that the sneezing and cough of the epidemic was very distressing. In two months from the date of the accident every wound had healed, and he had recovered the use of both arms." (P. 228.)

Mr. Paul announces his intention of bringing out this work quarterly, instead of monthly; and the first Number of the Journal, in its new shape, was to appear on the first Saturday in September. We wish it every possible success.

